# Antibiotic Resistance of Gram-Negative Bacteria from Wild Captured Loggerhead Sea Turtles

**DOI:** 10.3390/antibiotics9040162

**Published:** 2020-04-06

**Authors:** Monica Francesca Blasi, Luciana Migliore, Daniela Mattei, Alice Rotini, Maria Cristina Thaller, Rosa Alduina

**Affiliations:** 1 Filicudi Wildlife Conservation, Location Stimpagnato Filicudi, 98055 Lipari (Me), Italy; 2 Department of Biology, Tor Vergata University of Rome, 00133 Rome, Italy; luciana.migliore@uniroma2.it (L.M.); alice.rotini@gmail.com (A.R.); thaller@uniroma2.it (M.C.T.); 3 Department of Environment and Health, National Institute of Health, 00161 Rome, Italy; daniela.mattei@iss.it; 4 Italian Institute for Environmental Protection and Research, 00144 Rome, Italy; 5 Department of Biological, Chemical and Pharmaceutical Sciences and Technologies (STEBICEF), University of Palermo, 90028 Palermo, Italy

**Keywords:** antibiotic resistance, *Caretta caretta*, marine habitats, bacterial ecology, feeding, marine microbial ecology, marine bacteria, Mediterranean Sea

## Abstract

Sea turtles have been proposed as health indicators of marine habitats and carriers of antibiotic-resistant bacterial strains, for their longevity and migratory lifestyle. Up to now, a few studies evaluated the antibacterial resistant flora of Mediterranean loggerhead sea turtles (*Caretta caretta*) and most of them were carried out on stranded or recovered animals. In this study, the isolation and the antibiotic resistance profile of 90 Gram negative bacteria from cloacal swabs of 33 Mediterranean wild captured loggerhead sea turtles are described. Among sea turtles found in their foraging sites, 23 were in good health and 10 needed recovery for different health problems (hereafter named weak). Isolated cloacal bacteria belonged mainly to Enterobacteriaceae (59%), Shewanellaceae (31%) and Vibrionaceae families (5%). Although slight differences in the bacterial composition, healthy and weak sea turtles shared antibiotic-resistant strains. In total, 74 strains were endowed with one or multi resistance (up to five different drugs) phenotypes, mainly towards ampicillin (~70%) or sulfamethoxazole/trimethoprim (more than 30%). Hence, our results confirmed the presence of antibiotic-resistant strains also in healthy marine animals and the role of the loggerhead sea turtles in spreading antibiotic-resistant bacteria.

## 1. Introduction

In the last century, the abuse and misuse of antibiotics led to the alarming emergence of antibacterial-resistant (ABR) strains that represent an increasing threat for both human and animal health worldwide [1,2,3]. In recent years, the spread of ABR strains has been frequently reported in natural environments, i.e., wild animals, soils, seawater. Although aquatic environments are characterized by low bacterial concentrations in respect to others, such as soil or animals, several studies have demonstrated an increase of resistance traits in these environments as well [4]. Thus, marine animals, which are completely immersed in their environment, can be environmental health indicators, reservoirs and spreaders of ABR strains [5,6,7,8,9,10]. Additionally, the isolation of ABR strains can be considered as a spoiling index of the habitat of these animals [11,12]. ABR from marine animals could enter into the food web by ingestion of contaminated meat or could be released during egg deposition of female animals [13].

**Figure 1:**
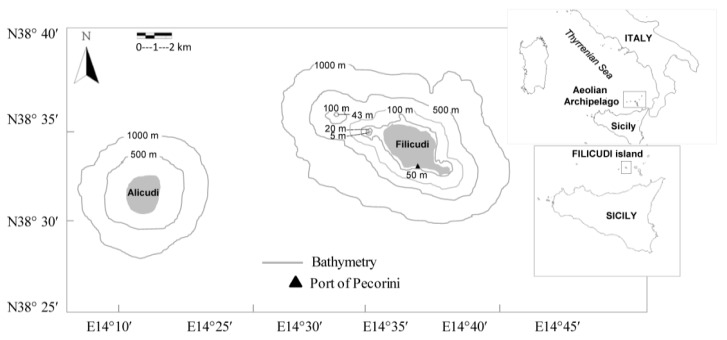
Sampling area of *Caretta caretta* in the Aeolian Archipelago (Southern Thyrrenian Sea, Sicily, Italy).

Sea turtles have been proposed as sentinel species in the Western Mediterranean Sea and carriers of ABR strains in marine habitats [10,13], for their migratory lifestyle [14,15] and their tendency to bio-accumulate contaminants, toxins and pathogens [16]. The loggerhead sea turtle (*Caretta caretta*) is the most common sea turtle species in the Mediterranean Sea, where it can encounter different threats of anthropogenic origin and undergo severe diseases and infections [16,17,18,19,20,21,22,23]. The Aeolian Archipelago, located in Southern Tyrrhenian Sea (Sicily, Italy) (Figure 1), is a volcanic area with adjacent extensive neritic and oceanic habitats within short distances [24]. Although it is not a usual nesting site for loggerhead sea turtles, it is considered an important foraging and over-wintering ground [25,26].

To date, a few studies investigated the microbial community associated with *Caretta caretta* by using culture-dependent methods [9,10,27,28,29] or metagenomic analysis [30,31,32], and even fewer studies were carried out on the antibiotic resistance of isolates [9,10,13,28]. All the latter studies collected samples from animals that needed to be recovered for several reasons (traumas, ingestion of fishing hooks, parasitosis, respiratory disorders) [9,10,13,28], or found dead along the seacoast [13]. Thus far, no studies exist to describe the microbial composition of healthy wild captured sea turtles in the Mediterranean Sea. It is likely that bacterial composition of recovered or dead animals can be influenced by several factors, i.e., health problems, conditions of recovery centres or therapies for treating illness conditions. Therefore, in this study, a screening for the Gram-negative bacteria in the cloacal swabs of wild captured Mediterranean loggerhead sea turtles from the Aeolian Archipelago was performed in order to evaluate the microbial composition and antibiotic resistance profile of bacteria isolated from wild healthy sea turtles.

## 2. Results

### 2.1. Loggerhead Turtles’ Size and Health Status

**Figure 2:**
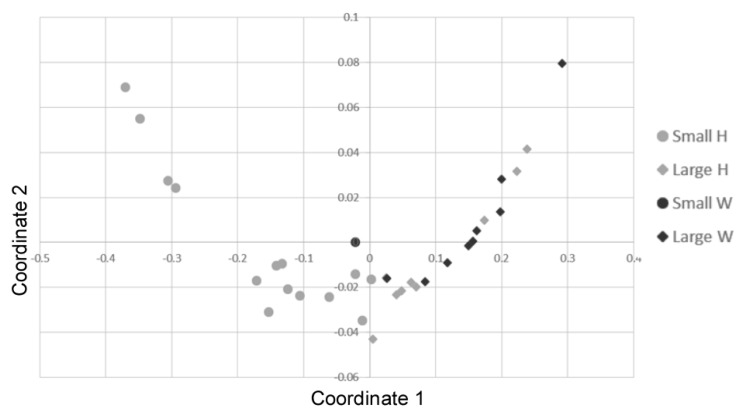
Non-Metric Multidimensional Scaling of body size distribution in the loggerhead turtles (parameters: CCL, curved carapace length and CCW, Curved Carapace Width) using the Bray-Curtis coefficient. Different size classes and health status are reported: small size = circle; large size = rhombus; heathy (H) = grey; weak (W) = black. Stress = 0.009511.

The 33 individuals had a mean curved carapace length (CCL) = 51.5 ± 10.7 cm ranging from 31 to 76 cm and a mean weight = 25.4 ± 21.4 kg, that is the developmental stage typically found in juvenile loggerhead turtles from the western Mediterranean [33]. In particular, the CCL of the 33 captured turtles was ≤50 cm in 15 individuals and >50 cm in 18 individuals. In this study, 23 individuals were considered as healthy while 10 individuals were weak. The latter were the sea turtles with a problem (hook, plastic presence, gastrointestinal occlusion, collision with a boat) that needed a recovery period. A significant correlation was found among the different body measurements recorded (Spearman’s rank; R = 0.95, *p* < 0.001). Consequently, the *n*-MDS analysis was performed on CCL and curved carapace width (CCW) measures only [33], showing a homogeneous distribution of individuals (Figure 2). However, a statistical difference in the mean size (CCL) between healthy and weak individuals was found; particularly, although the weight did not vary (Kruskal Wallis; *p* > 0.005), weak individuals (i.e., 1 small-size and 9 large-size; mean CCL = 60.5 ± 12.5 cm) were larger than the healthy ones (i.e., 14 small-size and nine large-size individuals; mean CCL = 47.6 ± 10.1 cm) (ANOSIM; R = 0.2998, *p* < 0.005).

### 2.2. Cloaca Associated Bacteria

**Figure 3:**
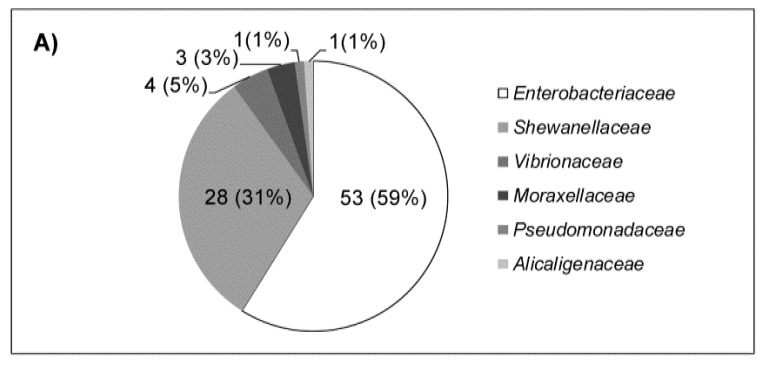

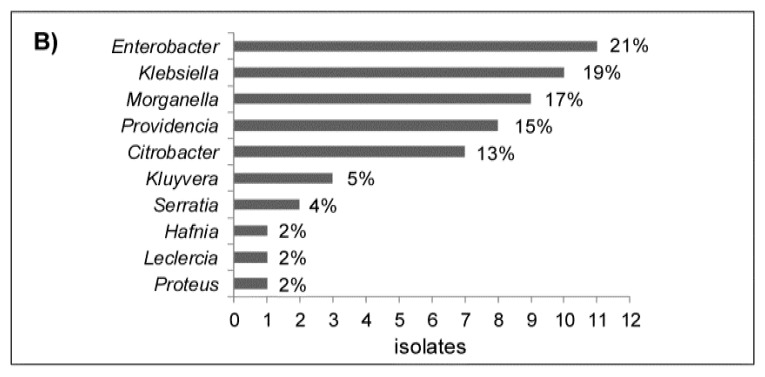
Percentage of cloaca-associated bacterial isolates (*n* = 90) for each family (**A**) and of bacterial isolates (*n* = 53) for each genus (**B**) of the Enterobacteriaceae family is reported.

In total, 90 enteric isolates were isolated from the cloacal swabs of sea turtles belonging mostly to Enterobacteriaceae (59%) and Shewanellaceae (31%) families while Vibrionaceae (5%), Pseudomonadaceae (i.e., *Pseudomonas* spp.), Alcaligenaceae (i.e., *Achromobacter animicus*) and Moraxellaceae (i.e., *Acinetobacter* sp.) were less represented, with 3%, 1% and 1%, respectively (Figure 3A). Among the Enterobacteriaceae family (Figure 3B), the most frequent genera were *Enterobacter*, mainly *E. cloacae* complex, and *Klebsiella*, mainly *K. oxytoca*. All the *Citrobacter* isolates belonged to the *C. freundii* group. The only *Proteus* isolate belonged to the described *Proteus cibarius* species associated to seafood [34]. All the *Shewanella* strains belonged to the *S. algae/S. haliotis* cluster, whilst the three *Vibrio* isolates were identified as three species: *V. alginolyticus, V. anguillarum* and *V. furnissii*.

**Figure 4:**
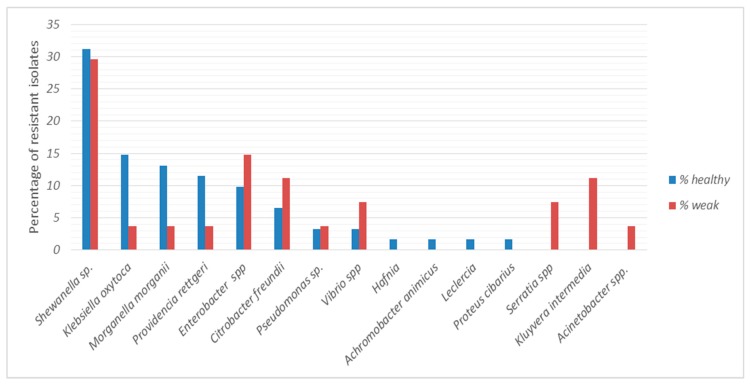
The relative percentage of bacteria identified in cloacal swabs from healthy (*n* = 23) and weak (*n* = 10) turtle cloacal samples is reported.

Eight bacterial genera were found in cloacal samples from both healthy and weak turtles, but in some cases their relative percentages varied. In particular, *Shewanella* sp., *Enterobacter* spp., *Citrobacter freundii*, *Pseudomonas* and *Vibrio* were equally distributed, while *Klebsiella*, *Morganella* and *Providencia* were more abundant in cloacal samples of healthy turtles. In contrast, *Hafnia*, *Achromobacter*, *Leclercia* and *Proteus cibarius* were found only in healthy turtles and *Acinetobacter*, *Kluyvera intermedia* and *Serratia* only in weak turtles (Figure 4).

### 2.3. Antibiotic Resistance

**Figure 5:**
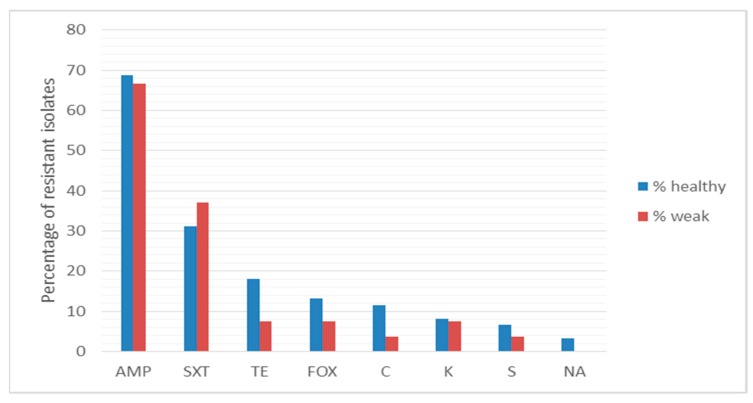
Percentage of antibacterial resistance of cloacal isolates from healthy and weak turtles. AMP, ampicillin; SXT, sulfamethoxazole/trimethoprim; TE, tetracycline; FOX, ciprofloxacin; CLO, chloramphenicol; KAN, kanamycin; STR, streptomycin, NAL, nalidixic acid.

The resistance profile of all the isolates to 10 antibiotics was determined. In total, 74 strains were endowed with one or more resistance phenotype. Among the bacterial isolates, 38% displayed resistance towards one antibiotic, mainly ampicillin or sulfamethoxazole/trimethoprim; 31% were resistant to two antibiotics, mainly ampicillin and sulfamethoxazole/trimethoprim, ciprofloxacin, or tetracycline. Furthermore, 8%, 2% and 3% of isolates were resistant to three, four and five antibiotics, respectively. The comparison of resistance profile among bacterial strains isolated from healthy and weak sea turtles is reported in Figure 5. Almost 70% and more than 30% of bacterial isolates from both healthy and weak animals were resistant to ampicillin and sulfamethoxazole/trimethoprim, respectively. The resistance to tetracycline, ciprofloxacin, chloramphenicol, kanamycin, streptomycin and nalidixic acid was registered at a lesser extent (from 18% to 3%). In addition, no significant difference in antibiotic resistance was detected in cloacal isolates from healthy and weak turtles (Kruskal-Wallis; *p* > 0.05). No resistant isolates to gentamycin and amikacin were found.

## 3. Discussion

In this study, we report for the first time the isolation and the antibiotic resistance profile of Gram-negative bacteria, from wild free-living loggerhead sea turtles in the Mediterranean Sea. A few studies have previously described the microbiota associated with *Caretta caretta* turtles [30,31,32], but the analysis was carried out on fecal samples of animals which experienced from few to many days in specialized centers [30,31,32], or on the intestinal samples of dead animals [30]. These studies revealed the gut microbial composition of loggerhead sea turtles by using the metagenomics approach, but results could be biased by the health conditions of the animals, by conditions, treatments or therapies in the recovery centers. Metagenomics allows a deep understanding of microbial community composition but also includes DNA of non-viable bacteria. In this study, the culture-dependent approach was followed, since we were also interested in the antibacterial resistance profile of isolates. In the last decades, the whole world witnessed an alarming increase of ABR strains from wild and pristine environments, including seawaters and marine species, which should not be exposed to antibiotics. Notwithstanding, several factors contributed to this phenomenon, primarily the selective effect of the antibiotic contamination in marine compartments (water and sediments) as a consequence of the wide use of antibiotics in aquaculture and intensive farms [35], and the discharge of wastewater containing antibiotics, due to the abuse and misuse in human medicine [1].

In addition, this study was finalized to search for aerobic Gram-negative bacteria, since they are usually more abundant and frequent in marine sea turtles [36]. In coastal seawater, these microorganisms are also present (and indicators) in the case of anthropic impact; hence, the search for these microorganisms can give an indication of the reciprocal risk (*Caretta*-humans) of spreading pathogens and antibiotic resistance. In addition, Gram-negative bacteria, considered as opportunistic pathogens, can become harmful if stressful conditions (e.g., poor water quality, trauma, over-crowding) occur with a consequent decrease of immune response [37,38]. Our results showed the prevalence of Enterobacteriaceae (59%) and Shewanellaceae (31%). These percentages are lower than those reported in previous studies that found 90% of Enterobacteriaceae and 40% of Shewanellaceae in cloacal swabs of sea turtles recovered from near shore environments of the Tyrrhenian sea and kept at a recovery center [10]. *Shewanella* sp. was the most abundant isolate in our samples (31% and 30% in healthy and weak sea turtles), while *Citrobacter* and *Aeromonas* were the most abundant in other reports [10,13]. This difference could be ascribed to different health conditions or origin of sea turtles or to diverse microbial composition of Mediterranean Sea (the present survey was carried out one and four years before the cited reports). The bacterial species belonging to Enterobacteriaceae and Shewanellaceae found in this study are frequently associated to the microbiota of marine animals, including sea turtles [9,30]. Enterobacteriaceae are frequently found in the gut flora of reptiles [39,40]. Previously known as commensal bacteria, today Enterobacteriaceae are considered as cause of serious illness worldwide, mainly in immunocompromised patients [41], and suggested as potential secondary invaders of infections in marine animals [42]. *Enterobacter* spp. has been previously isolated from the swabs of green turtles (*Chelonia mydas*) affected by fibropapillomas [43] and from the oviductal fluid of both *Chelonia mydas* [44] and loggerhead sea turtles [9]. *Citrobacter freundii* is a well-known captive sea turtle pathogen, responsible for cutaneous ulceration, sloughing skin septicemia, dehydration and death [16,45]. *Klebsiella oxytoca* was already reported in other Mediterranean areas associated with loggerhead turtles [9,27]). *Shewanella* spp. have been isolated from both marine [46], freshwater fish [47] and molluscs [48]. *S. putrefaciens*, described as a pathogen of marine organisms, is a species commonly isolated from the cloaca of loggerhead sea turtles also in the Mediterranean Sea [9,10,28] as well as reported in this study.

Comparison between the microbial composition of healthy and weak sea turtles did not reveal differences in term of bacterial genera; indeed, a bacterial core constituted by *Shewanella* sp., *Klebsiella oxytoca, Morganella morganii, Providencia rettgeri, Enterobacter* spp., *Citrobacter freundii, Pseudomonas* sp. and *Vibrio* spp. was found; however, some differences in terms of prevalence of some taxa were registered. In fact, *Enterobacter, Citrobacter, Providentia* and *Shewanella* were more frequently found in weak turtles; this result is in accordance with previous reports on the microbial composition of sea turtles injured by anthropogenic threats, or feeding on longlines bait and on marine debris [9,10,13,27,28]. The vulnerability of loggerhead turtles to be colonized in their gut by aerobic heterotrophic bacteria may be related to different stress conditions due to their illness or a debilitated health status that may result in an altered metabolic state [49]. For example, the long residence time of marine debris in the gastrointestinal system might result in dietary dilution [49] increasing significantly the absorption of bacteria as a consequence of a reduced ability to recruit food sources. However, other reasons can be hypothesized. First, larger turtles, which are known to feed at higher trophic levels [15,33], might preferentially feed on prey items in neritic waters and, consequently, be more exposed to anthropogenic contaminations. Indeed, in our study, the mean CCL of weak individuals was higher than that of healthy ones as the adult turtles are known to be more frequently injured by different anthropogenic threats [25,26]. Secondly, variations in diet (i.e., trophic differences) and/or food opportunity could take place among individuals in different areas, since loggerhead sea turtles are migratory species and opportunistic predators [26,49,50]. These considerations suggest caution in the interpretation of the results on the gut microbial communities, when animals with different health conditions are compared. As a matter of fact, less differences of bacterial composition between healthy and weak animals in this study were registered compared to the differences with other reports on stranded, recovered or dead sea turtles. Four and three bacterial species were exclusively present in healthy and weak sea turtles, respectively. In particular, *Acinetobacter*, *Kluyvera intermedia* and *Serratia* were found only in weak turtles while *Hafnia*, *Achromobacter*, *Leclercia* and *Proteus cibarius* only in healthy turtles. The low number of isolates per bacterial species cannot lead to speculation on their presence and their association with animal health conditions. In addition, we cannot exclude an accidental presence of these bacteria not due to a stable symbiosis.

To our surprise, we found larger differences between the antibiotic resistance percentage of this study and other studies than between healthy and weak turtles of the present survey. Indeed, bacterial isolates were resistant to the beta-lactam antibiotic ampicillin (~70% in both healthy and weak animals) at a lower extent than bacteria isolated from cloacal swabs of free-living animals in a previous report (94.7%) [10]. Except for resistance to trimethoprim-sulfamethoxazole, that was higher in the cloacal isolates of this study (31% and 37% in healthy and weak animals, respectively) than in the latter report (22.7%), a less frequency of resistant phenotype was found. Precisely, in this survey, the percentage of isolates resistant to chloramphenicol (11% and 4% of isolates from healthy and weak animals), to ciprofloxacin (13% and 7%), to nalidixic acid (3% in both healthy and weak animals), to streptomycin (7% and 4%) and to tetracycline (18% and 7%) was lower than the resistance percentage (39%, 30%, 30%, 37% and 71%) found in other reports on stranded, recovered or dead sea turtles [9,10,28]. In a more recent report [13] on stranded and dead sea turtles, only resistance to tetracycline (19%) was similar. Based on these results, we can surmise that the differences of antibiotic resistance percentage could be dependent upon altered health conditions or diverse origin and age of sea turtles or to an increased contamination of Mediterranean Sea (this survey was carried out one or four years before the cited reports) or to methods adopted in different laboratories. Even if we cannot rule out an intrinsic natural resistance of some bacterial species, the diffusion of antibiotic-resistant strains and the corresponding resistance genes is worrying for marine environments and, consequently, for human health. Therefore, this study confirms *Caretta caretta* as a carrier of ABR strains for its migratory life cycle and highlights the importance to carry out microbiome analysis on healthy individuals.

## 4. Materials and Methods

### 4.1. Sample Collection

The cloacal samples were collected by dedicated boat surveys from March to December 2014 as previously described [25,26]. The loggerhead turtles (33 individuals) were temporarily captured by hand in their foraging grounds to take body measurements using standard methods [33] and to collect cloacal samples [12], and then released at sea or brought in the rescue centre if severe injuries, diseases or problems were detected. The weight (kg), curved carapace length and width (CCW, cm), plastron length and width (cm), head and tail length (cm) were recorded for each turtle. The CCL notch-to-tip was recorded from the nuchal scute notch to the tip of the supracaudal scute, as described [33]. The loggerhead turtles were classified based on CCL more or less than 50 cm (small size, CCL ≤ 50 cm; large size, CCL > 50 cm). Sexual maturity in loggerhead individuals was not recorded but 70 cm is generally considered as the minimum CCL for Mediterranean loggerheads to be classified as adult [51]. Consequently, the 33 sampled turtles ranged from small juvenile to adult stage.

In addition, the health status was outlined according to the “Guidelines for the recovery, rescue, foster care and management of sea turtles for the rehabilitation and handling for scientific purposes” (http://www.isprambiente.gov.it/en/publications/handbooks-and-guidelines/guidelines-for-the-recovery-rescue-and-management-of-sea-turtles-for-the-purposes-of-rehabilitation-and-for-scientific-purposes). In particular, loggerhead turtles with detectable injuries were subjected to veterinary examination for establishing the likely cause of rescue, based on a complete external examination. The health status of the loggerhead turtles was evaluated according to the following parameters: (1) their inability to swim and dive, (2) thinness, weakness and deficient response to stimuli due to clear evidence of long-lasting injuries or severe diseases, (3) massive plastic found in faeces or evidences of severe boat collision or longline hook ingestion. A cause of rescue was assigned only if clear injuries/diseases were evident on the turtle body at sea or during their stay in the rescue centre (e.g., ingested longline hook, gastrointestinal occlusion due to massive debris ingestion or traumatic diseases due to collision with boats). Hence, the loggerhead turtles were thus classified as good health (H) or weak (W, live turtles with detectable injuries). All the cloacal samples were collected before the recovery at the rescue centre. Collection of samples was conducted following the International Council for Laboratory Animal Science Ethical Guidelines and was authorized by the Italian Ministry of Environment (PROT. N° 0001735, 02-02-2010; renewal: PROT N° 0006876, 25-01-2013).

### 4.2. Cloaca Associated Bacteria Analysis

The cloacal samples were collected by using sterile swabs (BD 440476; Becton, Dickinson, France S.A.) that were immediately shipped to the laboratory within 48 h from the collection and kept cool under darkness. Bacteria were isolated by a direct plating method [52] of the cloacal swabs on MacConkey agar plates. The plates were incubated for 24–48 h at 25 °C. One colony for each observed morphology was picked up, checked for purity, stored in Brain Heart Infusion (BHI; Difco) with 20% glycerol at −70 °C until further examination. Identification was performed by means of conventional and molecular analyses as previously described [53,54,55]. Briefly, 16S rDNA amplification and sequencing, universal primers, Com1 (forward, 5′-CAGCAGCCGCGGTAATAC-3′) and Com2 (reverse, 5′-CCGTCAATTCCTTTGAGTTT-3′), were used to amplify a 407 bp fragment encompassing the phylogenetically highly variable regions, V4 and V5 of the 16S rRNA gene. Amplification reactions were carried out in a 100 μL volume with 100 pmol of each primer in GoTaq Green Master Mix buffer (Promega, Madison, WI, USA) and 2 μL of the DNA solution in TE buffer diluted 1:30. The following cycling conditions were used: 3 min at 94 °C, 30 cycles (one cycle consists of 1 min at 94 °C, 1 min at 50 °C, and 70 s at 72 °C), and 5 min at 72 °C. The amplification products were purified and sequenced by Macrogen Europe (The Netherlands) and then the sequences were identified using BLAST program (http://blast.ncbi.nlm.nih.gov/Blast.cgi).

### 4.3. Antibiotic Susceptibility Test

Antimicrobial resistance profiles were studied by using the Kirby Bauer technique with the following antibiotics: ampicillin (10 μg), amikacin (30 μg), chloramphenicol (30 μg), ciprofloxacin (5 μg), gentamycin (10 μg), kanamycin (30 μg), nalidixic acid (30 μg), streptomycin (10 μg), sulfaprim (trimethoprim-sulfamethoxazole; 25 μg) and tetracycline (30 μg), as described elsewhere [56]. Antimicrobial disks were obtained from Oxoid (United Kingdom). Interpretation of results was carried out by referring to the Clinical & Laboratory Standards Institute (CLSI), 2018 range.

### 4.4. Statistical Analysis

The Spearman Rank was used to test correlation among different body measurements. The n-MDS (non-Metric Multidimensional Scaling) test, with the Bray-Curtis coefficient, was used to get a graphical representation of the morphological data distribution and to evaluate a possible relationship between morphometric data (reference parameters: CCL and CCW) and turtle health status (H or W). Multivariate non-parametric analysis of similarities (ANOSIM) on Euclidean distances was used to assess differences in body size and weight, between H and W groups. Normal distributions of data were checked using Shapiro-Wilks tests and the homogeneity of variances using Levene’s test. Kruskal-Wallis was also used to compare the resistance patterns of the bacterial isolates between healthy and weak turtles. All the analyses were performed using PAST v. 3.1 software (https://folk.uio.no/ohammer/past/).
